# Effects of encapsulated algae oil supplements on the production of docosahexaenoic acid–enriched milk in mid-lactation dairy cows

**DOI:** 10.3168/jdsc.2025-1004

**Published:** 2026-03-19

**Authors:** Yan Li, Hongling Jiang, He Zheng, Junhong Wang, Hongyun Liu

**Affiliations:** 1 College of Animal Sciences, Zhejiang University, Hangzhou 310058, China; 2 College of Animal Sciences, Xinjiang Agricultural University, Urumqi 830052, China; 3 Kemin (China) Technologies Ltd., Zhuhai 519040, China

## Abstract

This study was conducted to assess the effects of encapsulated algae oil (EAO) supplementation on the production of docosahexaenoic acid (DHA)-enriched milk in mid-lactation dairy cows. A total of 60 mid-lactation dairy cows were randomly assigned to 4 treatment groups, comprising the addition of EAO (DHA >17%) at 0, 60, 120, and 180 g/d per head (equivalent to 0, 10, 20, and 30 g/d DHA). The experiment lasted for 8 wk, with an additional 10-d prefeeding period. Compared with the control group, the ECM and fat content were increased in 10 g/d DHA group, but decreased with the 20 g/d and 30 g/d DHA levels. No significant treatment effect was observed on DMI and milk composition. The content of milk DHA exceeded 100 ppm (10 mg/100 mL) by the fifth week when 30 g/d DHA was administered. The conversion rate for all groups exceeded 10%, whereas the group supplemented with 10 g/d DHA exhibited the highest level. No significant differences were found in SFA or MUFA levels. In contrast, PUFA, including DHA, significantly increased with the EAO supplementation. In addition, compared with raw milk, the DHA content of pasteurized milk decreased by 8.39%, and UHT milk decreased by 25.64%. In summary, EAO supplements significantly increased the milk DHA content without influencing the overall milk performance of dairy cows.

## Highlights

• Encapsulated algae oil supplements linearly improved the DHA content in milk.
• The milk DHA content exceeded 10 mg/100 mL by the fifth week when 30 g/day DHA was supplemented.
• The DHA content in pasteurized and UHT milk was decreased under thermal processing.

## Graphical Abstract

**Figure:**
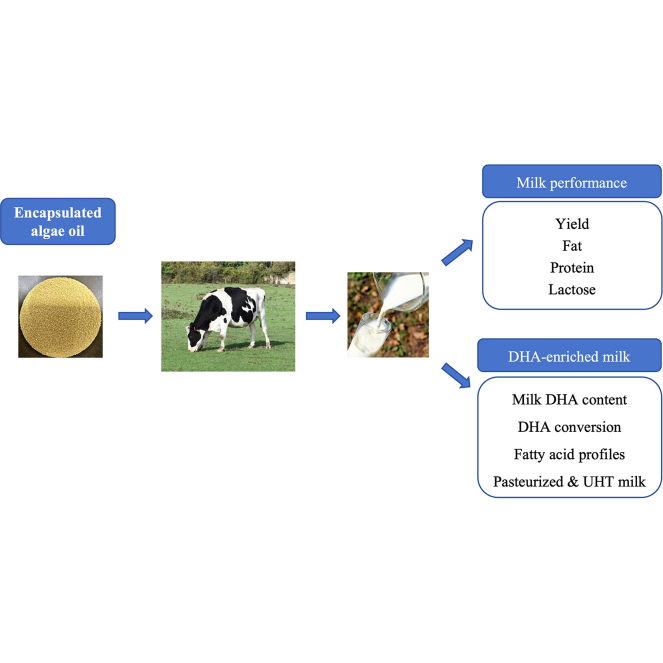
**Summary:**Docosahexaenoic acid (DHA) plays a crucial role in the cognitive development of infants and in mitigating cardiovascular diseases in the elderly. However, because the body cannot fully synthesize DHA, it must be obtained through diet. In this study, we explored the effects of encapsulated algae oil (EAO) supplementation on the production of DHA-enriched milk in dairy cows. The milk DHA content exceeded 10 mg/100 mL when 30 g/day DHA was administered, and the conversion rate for all groups exceeded 10%. The concentrations of polyunsaturated fatty acids, particularly DHA, demonstrated a significant increase with EAO supplementation. In addition, compared with raw milk, the DHA content of pasteurized milk decreased by 8.39%, and ultra-hightemperature (UHT) milk decreased by 25.64%. In conclusion, EAO supplements demonstrated the ability to improve the DHA-enriched milk significantly without influencing the overall milk performance of dairy cows.

As a PUFA, docosahexaenoic acid (**DHA**) critically supports infant cognitive maturation (Birberg-Thornberg et al., 2006), as well as mitigating cardiovascular disease risks in geriatric populations (Breslow, 2006). However, because the conversion of α-linolenic acid (**ALA**) to DHA is estimated to be less than 1% in the body, DHA must be obtained through the diet (Domenichiello et al., 2015). Over recent decades, the rapid improvement in the quality of life for global citizens has led to a growing demand for high-quality milk enriched with additional nutritional benefits. A particular emphasis has been placed on the production of DHA-enriched milk. Although postprocessing DHA fortification enables milk enrichment, this method may present certain disadvantages compared with naturally DHA-rich milk due to oxidation of fish or algal oils, which may cause undesirable flavors (Wright et al., 1998). Therefore, it is crucial to find a cost-effective and practical method to produce natural DHA-enriched milk. Dietary integration of microalgae and soybean oil in ruminant nutrition enhances milk lipid profiles through depressed SFA concentrations and augmented bioactive constituents, notably CLA, ALA, and DHA (Gagliostro et al., 2018). Other research has investigated the potential of producing naturally DHA-enriched milk by adding extruded flaxseed and algae meal to dairy cow diets (Moate et al., 2013; Zachut et al., 2010). These studies found that although milk DHA content increased significantly, milk fat content decreased correspondingly. The observed milk fat depression likely stems from ruminal biohydrogenation of UFA, which inhibits mammary lipogenesis (Bauman and Griinari, 2003). Thus, we hypothesized that dietary integration of encapsulated algae oil (**EAO**) could modulate the profile of milk fatty acid and enhance milk DHA content. The principal focus of this study was to assess the effects of dietary EAO integration on the production of DHA-enriched milk in dairy cows.

Sixty lactating dairy cows were selected and blocked according to DIM (136.5 ± 17.5 d) and milk yield (34.89 ± 2.43 kg), followed by random allocation to 4 treatments in 15 blocks. All cows were fed a basal TMR diet supplemented with 0, 60, 120, 180 g/d of EAO individually (DHA content: 17%, equivalent to 0, 10, 20, 30 g/d DHA). The EAO was provided by Kemin (China) Technologies Ltd. (Zhuhai, China). Animals were housed in freestall barns, ad libitum access to feed and water. Milking routines were conducted 3 times daily (at 0600, 1300, and 1900 h), with an 8-wk feeding period and a 10-d prefeeding adaptation phase. The EAO was added twice daily at 0630 and 1330 h, sprinkled on the surface of the TMR and mixed to ensure complete intake.

Daily average DMI was calculated for each group by measuring the provided TMR and refusals every day. Samples of the TMR were collected weekly and dried in a forced-air oven at 65°C for 48 h to determine the content of DM. The samples were ground with a Wiley mill equipped with a 1-mm screen (Thomas Scientific, Swedesboro, NJ) for subsequent analysis of crude protein and ether extract (AOAC, 1990). The ADF and NDF contents were determined using an Ankom A200 fiber analyzer (Ankom Technology, Macedon, NY) with heat-stable amylase and sodium sulfite (Fisher Scientific, Waltham, MA), with the results expressed inclusive of residual ash (Van Soest et al., 1991). Milk samples were collected on a weekly basis. For each cow, a 50-mL milk sample was obtained, with samples collected across the 3 daily milking times as previously described (Wang et al., 2014). To preserve samples, 0.6 mg/mL potassium dichromate was added, and they were then stored at 4°C for the subsequent analysis. An automatic ultrasonic milk composition analyzer (Bentley Instruments, Chaska, MN) was employed to assess the content of fat, protein, lactose, SCC, and MUN. At the last week, additional milk samples (100 mL/cow) were collected from the 30 g/d DHA group for the processing of UHT milk (135°C, 4 s) and pasteurized milk (72°C, 15 s) using a Microthermics Pasteurizer (model LAB-25 EHVH, Microthermics, Raleigh, NC). The profiles of milk fatty acids were examined by GC with an Agilent model 7890B gas chromatograph (Agilent Technologies) equipped with a Quadrex capillary column (100 m × 0.25 mm, 0.20 μm) and a flame ionization detector. The initial column temperature was 140°C. The temperature was increased at 4°C/min to 220°C, where it was maintained. The carrier gas was hydrogen, flowing at a linear velocity of 1.0 mL/min. The injection volume was set at 1 μL, and the split ratio was configured at 1:80 (GB5009.168-2016, China).

All data were performed with the MIXED procedure in SAS (version 9.2, SAS Institute Inc.). The experimental design adopted a randomized block structure with repeated measures, where treatment and week were defined as fixed effects and individual cow served as the random effect. Contrasts were developed to assess treatment effects and varying levels of EAO supplementation, using orthogonal polynomials to accommodate the unequal spacing of EAO levels. Group differences were analyzed by Tukey's multiple range test, with data presented as LSM and SEM. Significance was determined at *P* ≤ 0.05, and values of 0.05 < *P* ≤ 0.10 were considered to indicate trends.

Dietary fat supplements usually demonstrate varied effects on DMI, and these discrepancies could be influenced by several factors such as supplement formulation (physical form, odor, palatability), dosage parameters (concentration, duration), lactation stage, and basal diet composition (Moallem, 2018). In our study, incremental EAO inclusion (0, 60, 120, 180 g/d) elevated daily DHA provision to 0, 10, 20, and 30 g/cow, respectively. These doses were selected based on prior evidence linking high-dose DHA (>30 g/d) to reduced milk fat synthesis and impaired animal performance (Boeckaert et al., 2008; Moate et al., 2013; Thanh and Suksombat, 2015). Notably, our results align with Till et al. (2019), showing no DMI suppression at ≤24 g/d DHA. However, Moate et al. (2013) reported dose-dependent DMI declines (6% at 50 g/d; 11% at 75 g/d). Consequently, it appears that supplying EAO at up to 30 g/d can be achieved without adversely affecting intake. Compared with the control group, 10 g/d DHA significantly improved ECM (*P* = 0.02), whereas other DHA levels were observed an opposite trend. The content of milk, MUN and SCC showed no significant differences among all groups. Raw milk exhibited no significant differences among all groups, whereas ECM and fat yield displayed a tendency to decrease linearly (Table 1). These results corroborate Moate et al. (2013), who observed a reduction in ECM with algal meal supplementation while maintaining stable milk yield. Conversely, a reduction in milk yield associated with microalgae inclusion was observed, which was also consistent with a decrease in milk fat content (Vanbergue et al., 2018). Additionally, the supplementation of rumen-protected microalgae has been associated with milk fat depression (Glover et al., 2012), potentially due to the function of coating materials. Notably, our study did not exhibit such adverse effects, which may be attributed to the encapsulation materials that effectively protect DHA and prevent its degradation in the rumen.

**Table 1:** Effects of dietary EAO supplements on milk performance in dairy cows

Item	DHA, g/d				SEM	*P-*value^1^		
	0	10	20	30		T	L	Q
DMI, kg/d	20.88	21.43	21.52	21.58	1.13	—	—	—
Milk yield, kg/d								
Raw	34.85	35.60	35.03	35.13	0.43	0.36	0.84	0.30
ECM^2^	30.83^ab^	32.75^a^	29.89^b^	29.88^b^	0.74	0.02	0.09	0.20
Fat	1.03^ab^	1.12^a^	0.98^b^	0.98^b^	0.06	0.04	0.11	0.22
Protein	1.11	1.18	1.14	1.14	0.03	0.30	0.77	0.13
Lactose	1.81	1.87	1.75	1.73	0.07	0.15	0.09	0.39
Content, g/100 g								
Fat	3.15	3.18	3.00	2.95	0.15	0.63	0.24	0.80
Protein	3.46	3.32	3.54	3.40	0.09	0.09	0.95	0.97
Lactose	5.26	5.25	5.20	5.20	0.04	0.36	0.11	0.85
MUN, mg/dL	14.38	14.05	13.95	13.92	0.70	0.91	0.50	0.75
SCC, 10^3^/mL	72.12	84.75	79.48	70.43	1.84	0.85	0.86	0.41

a,b Means within a row with different superscripts are significantly different (*P* < 0.05).

1 T = treatment; L = linear; Q = quadratic.

2 ECM (kg/d) = 0.3246 × milk yield (kg/d) + 13.86 × milk fat yield (kg/d) + 7.04 × milk protein yield (kg/d); Orth (1992).

Encapsulated algae oil supplementation did not significantly alter the levels of SFA and MUFA, but a notable increase was observed in PUFA. Regarding individual fatty acids, in contrast to control group, a linear decrease was shown in C6:0, C8:0, C18:0, C18:1, and C20:4, whereas C14:1, C16:0, C16:1, C18:2, C18:2 *trans*-10,*cis-*12 (CLA), C22:5, and C22:6 (DHA) showed a linear increase (*P* < 0.05; Table 2). Biohydrogenation-derived fatty acid intermediates, particularly *trans*-10,*cis*-12 CLA, are well-documented inhibitors of milk fat synthesis (Peterson et al., 2003; Hussein et al., 2013), though other intermediates may contribute synergistically (Chilliard et al., 2001). Rumen supplementation with PUFA-rich oils or biohydrogenation intermediates suppresses both de novo fatty acid synthesis and mammary uptake of circulating lipids (Hussein et al., 2013), aligning with our observations. For instance, diets supplemented with fish oil or microalgae reduced mammary sterol regulatory element-binding protein (SREBP) expression by 15% compared with controls (Vahmani et al., 2013), further supporting PUFA-mediated transcriptional regulation. Although *trans*-10,*cis*-12 CLA exhibits potent antilipogenic effects (Bauman and Griinari, 2003; Lock et al., 2006), our study revealed its linear accumulation alongside declining milk fat content and yield with EAO supplementation. Notably, marine lipid–induced milk fat depression often occurs independent of *trans*-10,*cis*-12 CLA fluctuations, implicating alternative isomers or fatty acids in the inhibitory mechanism.

**Table 2:** Effects of dietary EAO supplements on milk fatty acid profiles in dairy cows (% of the Σ)

Fatty acid^1^	DHA, g/d				SEM	*P*-value
	0	10	20	30		
C4:0	1.69	1.77	1.87	1.63	0.118	0.510
C6:0	1.45^a^	1.49^a^	1.33^ab^	1.18^b^	0.077	0.037
C8:0	0.97^a^	0.96^a^	0.87^ab^	0.76^b^	0.054	0.036
C10:0	2.38	2.27	2.11	1.88	0.140	0.102
C12:0	3.00	2.84	2.76	2.62	0.154	0.403
C13:0	0.09	0.08	0.08	0.07	0.007	0.196
C14:0	11.17	10.75	11.07	11.12	0.270	0.685
C14:1 *trans*	0.21	0.21	0.19	0.19	0.007	0.156
C14:1	1.06^b^	1.10^b^	1.31^ab^	1.49^a^	0.116	0.034
C15:0	1.00	0.93	1.01	1.01	0.046	0.550
C15:1 *trans*	0.24	0.26	0.23	0.30	0.018	0.025
C16:0	38.3^b^	39.54^bc^	40.48^ac^	41.82^a^	0.704	0.011
C16:1	1.80^b^	2.08^b^	2.52^b^	3.30^a^	0.270	0.003
C17:0	0.50	0.47	0.51	0.52	0.015	0.156
C18:0	9.67^a^	9.43^a^	9.20^a^	7.55^b^	0.416	0.003
C18:1	23.00^a^	21.9^ab^	20.52^b^	20.25^b^	0.630	0.020
C18:2	2.72^b^	2.99^b^	2.90^b^	3.14^a^	0.103	0.050
C18:2 *trans*-10,*cis*-12 (CLA)	0.02^b^	0.03^ab^	0.04^ab^	0.05^a^	0.003	0.050
C18:3	0.2	0.28	0.27	0.27	0.016	0.776
C20:0	0.13	0.13	0.13	0.13	0.003	0.847
C20:3	0.17	0.14	0.17	0.19	0.016	0.244
C20:4	0.19^a^	0.13^b^	0.11^b^	0.12^b^	0.010	<0.001
C20:5n-3 (EPA)	0.02	0.02	0.03	0.04	0.004	0.024
C22:5n-3 (DPA)	0.01^d^	0.04^c^	0.06^b^	0.07^a^	0.004	<0.001
C22:6n-3 (DHA)	0.01^d^	0.13^c^	0.28^b^	0.35^a^	0.019	<0.001
SFA^2^	70.31	70.66	71.42	70.28	0.724	0.651
MUFA^3^	26.31	25.60	24.76	25.54	0.707	0.489
PUFA^4^	3.38^b^	3.74^bc^	3.81^ac^	4.17^a^	0.132	0.003

a–d Means within a row with different superscripts are significantly different (*P* < 0.05).

1 EPA = eicosapentaenoic acid; DPA = docosapentaenoic acid; DHA = docosahexaenoic acid.

2 SFA = ∑ (C4:0, C5:0, C6:0, C10:0, C12:0, C13:0, C14:0, C15:0, C16:0, C17:0, C18:0, C20:0, C22:0, C23:0, C24:0).

3 MUFA = ∑ (C14:1 *trans*, C14:1, C15:1 *trans*, C16:1, C18:1).

4 PUFA = ∑ (C18:2, C18:3, C20:3, C20:4, C20:5, C22:5, C22:6).

The milk DHA content reached over 10 mg/100 mL by the fifth week with 30 g/d DHA supplementation (Figure 1A). The changes in conversion rate of dairy cows are depicted in Figure 1B, revealing efficiency of 16.33%, 13.75%, and 11.76% when 10 g, 20 g and 30 g DHA were supplemented, respectively. Compared with raw milk (Figure 1C), the DHA content of pasteurized milk decreased by 8.39% (11.90 vs. 10.90 mg/100 mL), and UHT milk decreased by 25.64% (11.90 vs. 8.85 mg/100 mL). In milk, thermal processing is the most common method of controlling microbes and extending shelf life. However, excessive heating causes undesirable alterations in milk, such as lipid oxidation and protein denaturation (Wang et al., 2024a). The current investigation revealed a decline in the DHA content of pasteurized and UHT milk, suggesting alterations in the milk's lipid composition. Fat globules are the form of fat found in milk. Triacylglycerols, which constitute the core of milk fat globules, are enveloped by a 3-layered biofilm referred to as milk fat globule membrane (**MFGM**; Wang et al., 2021). These membranes are made up of phospholipids, cholesterol, triglycerides, membrane-specific proteins, and enzymes (Wang et al., 2019). The dissociation of native MFGM proteins and the subsequent binding of denatured whey proteins to MFGM proteins are accelerated by thermal treatments exceeding 65°C (Wiking et al., 2022). Analysis of the SDS-PAGE patterns of major MFGM proteins in milk subjected to various heat treatments (direct steam heating [**DSH**], indirect steam heating [**ISH**], and pasteurization) revealed that DSH and pasteurization treatments preserve a higher proportion of major MFGM proteins. However, the increased thermal load from ISH treatment leads to significant denaturation of the primary MFGM proteins in milk, with up to 50.05% denaturation observed after a 135°C/5 s treatment (Wang et al., 2019; Yan et al., 2022; Wang et al., 2024b).

**Figure 1:**
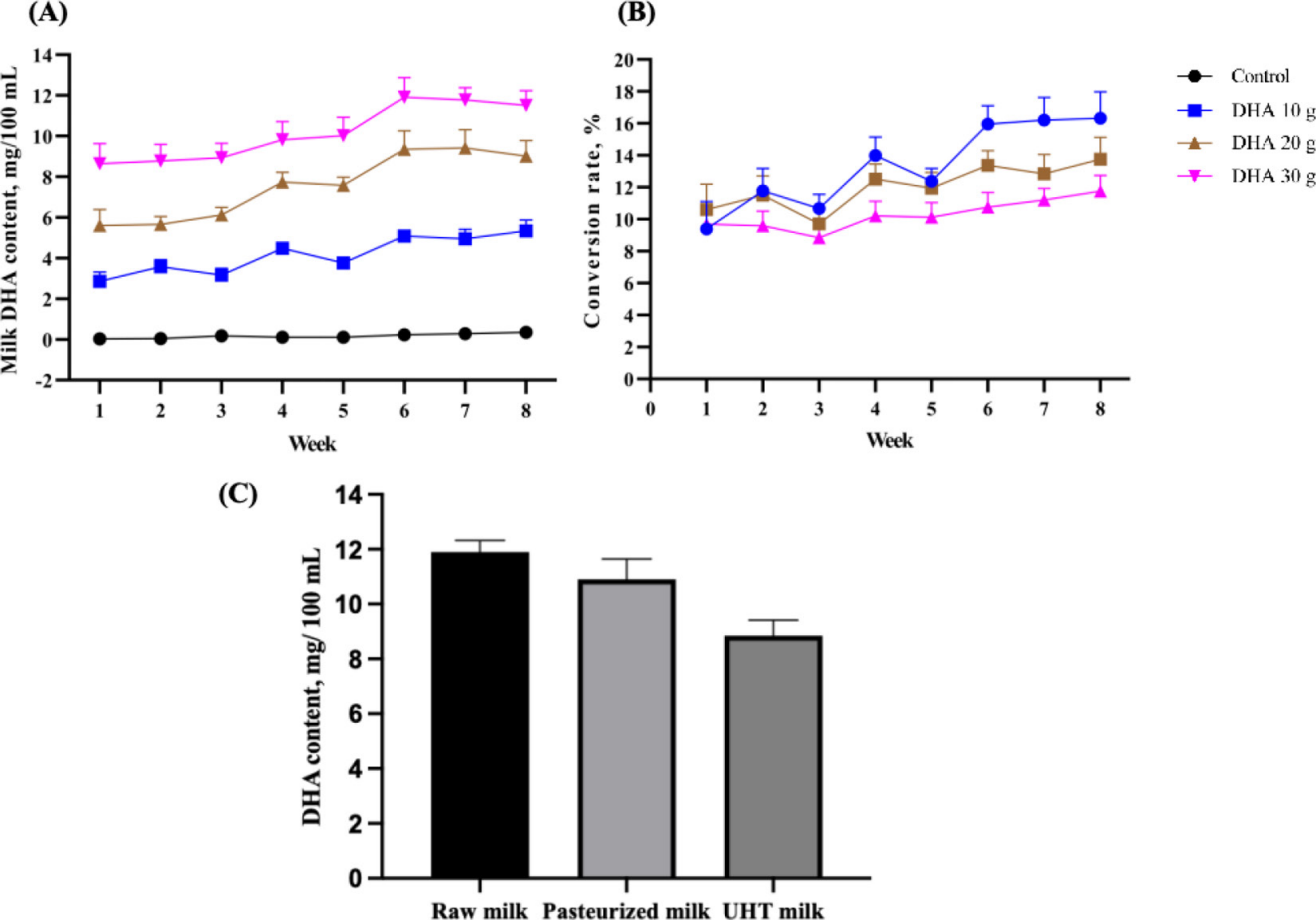
(A) Fluctuations in milk DHA content of dairy cows fed a basal diet supplemented with EAO. (B) Fluctuations in conversion rate of dairy cows fed a basal diet supplemented with EAO; conversion rate = milk DHA content/feed DHA content. (C) The DHA content of pasteurized and UHT-processed milk. Bars represent SEM.

Encapsulated algae oil supplementation in dairy cows could enhance the production of DHA-enriched milk without significantly affecting the milk performance. At the dose of 30 g/d DHA, the milk DHA content exceeded 10 mg/100 mL stably after 5 wk. In addition, the DHA content in pasteurized and UHT milk decreased after thermal processing, which is a key factor that should be considered for producing higher content DHA-enriched milk.
